# Effect of Topical Furosemide Efficacy on Reducing Bleeding and Quality of Surgical Field During Endoscopic Sinus Surgery in Patients with Chronic Rhinosinusitis

**DOI:** 10.22038/IJORL.2022.57445.2973

**Published:** 2022-09

**Authors:** Javaneh Jahanshahi, Mohammad Ghorbani, Farnaz Hashemian, Farshad Hashemian, Mojgan Sheikhi, Elham Khanlarzadeh

**Affiliations:** 1 *Department of Otolaryngology, School of Medicine, Hamadan, Hamadan, Iran* *.*; 2 *Department of Clinical Pharmacy, Pharmaceutical Sciences Branch, Islamic Azad University, Tehran, Iran.*; 3 *Student of Drug Control, Faculty of Pharmacy, Tehran University of* *Medical Sciences, Tehran, Iran.*; 4 *Department of Social Medicine, School of Medicine, Hamadan University of Medical Sciences, Hamadan, Iran.*

**Keywords:** Bleeding, Chronic rhinosinusitis, Endoscopic sinus surgery, Topical furosemide, Surgical field

## Abstract

**Introduction:**

Bleeding during endoscopic sinus surgery has an unfavorable effect on the surgical field and prolongs the time of surgery. In this study, we assessed the efficacy of topical furosemide on bleeding and the quality of the surgical field during endoscopic sinus surgery.

**Materials and Methods:**

In this clinical trial, 76 patients with chronic rhinosinusitis were selected for endoscopic sinus surgery and randomly assigned to two groups, topical furosemide (intervention) and normal saline (control). The intervention group received 20 micrograms of intranasal spray twice daily, and the control group received regular intranasal saline spray, similar to the intervention group. In addition, the quality of the surgical field (scoring by the BOEZAART grading system) and the amount of bleeding during surgeries were measured. All data were analyzed.

**Results:**

In the intervention and control groups, the mean surgical bleeding volume was 187.70± 24.79 and 229.21± 28.18 ml (P <0.001), the mean of Boezaart scale 2 and 3 (P <0.001) and the mean of surgical time were 106.53±14.67 and 126.63 ± 15.42 minutes (P <0.001), respectively. In patients of the intervention group with and without polyps, the mean surgery time was 99.56± 12.15 and 118.84 ±10.03 minutes (P <0.001), and the mean bleeding volume during endoscopic sinus surgery was 176.46 ± 22.58, 208.46 ±12.14 ml (P <0.001) respectively.

**Conclusions:**

Our findings showed that nasal, topical furosemide spray significantly reduced the amount of bleeding during endoscopic sinus surgery and time of the surgery and improved the quality of the surgical field.

## Introduction

Chronic rhinosinusitis (CRS) is a term used to describe a condition with chronic symptoms of inflammation or infection of the nose and sinuses (1). Chronic rhinosinusitis is divided into two types without polyps and with polyps. Initial treatment focuses on non-surgical pharmacological methods such as corticosteroids. However, even though medical treatment usually makes satisfactory control in most patients, some cases need surgery (2). 

Bleeding during endoscopic sinus surgery (FESS) remained a challenge for the surgeon and the anesthesiologist (3). 

Although extensive bleeding during endoscopic sinus surgery is rare, it is difficult for the surgeon to establish a desirable surgical field, and even minor bleeding by impairing endoscopic vision may increase complications such as CSF leak, blindness, diplopia, and damage to the internal carotid artery and also prolonging the time of surgery and even incomplete surgery (4). Therefore, many techniques have been proposed to improve the field of sinus surgery. Bipolar diathermy, packing, topical vasoconstrictors, and induced hypotension are the most commonly used techniques (5,6). In addition, using some drugs, such as oral corticosteroids, may reduce intraoperative bleeding before surgery, but some patients, such as diabetic, hypertensive, and glaucoma subjects, cannot use these drugs, so choosing an effective and safe drug can be a great help to a surgeon in controlling bleeding.

Furosemide is a loop diuretic and inhibitor of 2 CL_ K_NA cotransporters. 

That reduces prostaglandin E2 and F2a and inhibits cytokine release by reducing the secretion of arachidonic acid. As a result, inhaled furosemide does not have serious complications or diuretic effects (7). Furosemide at the epithelial level of the respiratory system reabsorbs sodium, followed by water and resulting in shrinkage of the polyp. The effects of furosemide on reducing edema and inflammation and thus reducing the size of polyps can reduce bleeding, especially in patients with contraindicated other drugs such as corticosteroids (7). It could be prescribed as an oral agent, intranasal spray, or intravenous infusion, with 60 minutes half-life.

If consumed through the nose, its onset and the peak effects are achieved within 10-15 minutes and 1-5 hours, respectively. Complications of furosemide include sodium excretion (with a probability of 0.84%), chlorine, and finally, water, hypotension, potassium depletion, and renal complications. All of them have been reported in therapeutic doses (at least 20 mg per day) over ten weeks. 

So far, limited studies have been worked on the inhaled effect of topical furosemide in reducing bleeding during endoscopic sinus surgery (3,8). Therefore, the present study was performed to determine the effect of intra-nasal furosemide on bleeding rate and field quality during FESS surgery in patients with chronic rhinosinusitis with and without polyposis.

## Materials and Methods

The research was performed in Besat Hospital of Hamadan University of Medical Sciences from December 2019 to February 2021 with the ID of the ethics committee ir.umsha.rec.1398.518. In this double-blind, randomized clinical trial, all patients referred to the otolaryngology clinic of Besat Hospital in Hamadan who were physically ASA1 and 2, according to AAO-HNS criteria, had chronic rhinosinusitis with or without polyposis; they did not respond to typical medical treatment and were candidates for FESS surgery.

The results of a clinical trial conducted by Baradaranfar et al. (8) reported that the mean ± standard deviation (SD) of intraoperative bleeding volume after the treatment was 262.15 ± 45.57 and 232.75 ± 56.08 in the Corticosteroid and furosemide groups, respectively. Therefore, we reached a sample of 38 for each group, a total sample size of 76 with an effect size of 0.6 and a type I error of 5 percent and 80 percent statistical power. After obtaining conscious and voluntary consent, they were assigned to the intervention or control group using a computer random number generator. Chronic rhinosinusitis with or without polyposis (primary or recurrent), noncompliance of patients to use corticosteroids, or with a medical contraindication of using corticosteroids, age 18-60 years, and normal PT, PTT, INR, BT, CT, and platelet count were inclusion criteria of the study. History of uncontrolled thromboembolic events, acute or chronic uncontrolled renal failure, receiving heparin 48 hours before surgery, receiving aspirin for three days before surgery, history of furosemide allergy, alcoholic liver disease, heart failure, having a cardiac stent, and having a sinonasal tumor were among the exclusion criteria of the study. All patients used nasal spray with unknown content (according to the start time of furosemide effect), one puff on each side of the nose twice a day, one week before the surgery date. Each puff of drug was equivalent to 20 micrograms of furosemide and 40 micrograms in total. This drug was made in Pharmacy Faculty at the Hamadan University of Medical Science. Normal saline was used in the control group. Because the study was double-blind, the drug and placebo were prepared in the same container by a pharmacist who had no relation to choosing patients. Therefore, the patients and the surgeon were unaware of its contents.

Due to randomization, patients with Polyposis were equally placed into two groups. Both groups, before surgery, underwent axial & coronal PNS CT SCAN and were also examined under a scoring endoscope and evaluated according to Lund-Kennedy criteria (9). The day before surgery, coagulation tests (PT, PTT, INR, BT, and CT) and electrolytes (in terms of the effect of furosemide on sodium and potassium) were measured for all patients.

The anesthesia method was assimilated for patients using midazolam, propofol, and atracurium and maintaining blood pressure. Before starting surgery, the phenylephrine-impregnated mesh was placed evenly on both sides of the nose of all patients for ten minutes, and a solution containing five cc of 1% lidocaine and 1.100,000 epinephrine was injected into the nasal mucosa, moderate arterial hypertension was recorded before surgery, and then surgery was started. In addition, the quality of surgical field vision at the end of surgery based on Boezaart Grading and also the amount of intraoperative bleeding at the end of surgery was recorded based on the amount of blood collected in the suction bottle (after deducting the volume of used normal saline) and the weight of blood- impregnated packs (which used during surgery).

Boezaart grading:

0- No bleeding (cadaveric conditions)

1 -Slight bleeding: no suctioning required

2- Slight bleeding: occasional suctioning required

3 -Slight bleeding: frequent suctioning required, bleeding threatens the surgical field a few seconds after suction is removed

4- Moderate bleeding: frequent suctioning is required, and bleeding threatens the surgical field directly after suction is removed

5- Severe bleeding: constant suctioning required; bleeding appears faster than can be removed by suction; surgical field severely threatened, and surgery usually not possible.

Also, mean arterial blood pressure was recorded during the surgery. During surgery, normal saline is given according to the patient's blood loss and weight. Also, the time of the surgery was recorded, including the start time until the end of packing.

The questionnaires were recorded in SPSS-16 software after completion. Descriptive information and qualitative data were expressed in tables, graphs, ratios, and percentages. Student t-test and chi-square were used to compare the variables of the intervention and control groups, Student t-test was used for the amount of bleeding during surgery, and the Mann-Whitney test was used for the quality of the surgical field. All data were considered significant at a 95% confidence level with P-value <0.05. 

## Results

In this study, which aimed to determine the effect of topical furosemide on reducing bleeding and improving the quality of the surgical field during endoscopic sinus surgery in patients with chronic rhinosinusitis, 75 patients were studied in the intervention (n = 37) and control (n = 38) groups. One patient in the intervention group was excluded due to a lack of patient cooperation. 

Patients with chronic rhinosinusitis undergoing endoscopic sinus surgery in the intervention and control groups were similar in age, previous history of surgery, gender, and endoscopic surgery evaluation parameters based on Lund-Kennedy were the same (Table 1). Mean arterial blood pressure was 12.10 ± 23 and 12.25 ± 25 mm Hg in the intervention and control groups, respectively. 

**Table 1:** Baseline characteristics in the two groups of intervention and control

**P-value**	**Treatment group**		**Variable **
	**control**	**Intervention **	
	Number (%)	Number (%)	Gender
	(65.8)25	(64.9)24	Male
0.933	(34.21)13	(35.1)13	Female
	(100)38	(100)37	Sum
	Number (%)	Number (%)	History of previous FESS
	(84.2)32	(78.4)29	Yes
	(15.8)6	(21.6)8	No
0.517	(100)38	(100)37	Sum
	Mean (Deviation standard)	Mean (Deviation standard )	Age
0.154	37.05 (11.99)	40.95 (11.38)	Year
	Number (%)	Number (%)	Evaluation score based on Lund-Kennedy scale
			Polyp
	(78.9)30	(62.2)23	Has
0.139	(21.1)8	(37.8)14	Doesn’t have
			Edema
0.602	(92.2)35	(94.5)35	Has
	(7.8)3	(5.5)2	Doesn’t have
			Secretions
0.592	(84.3)32	(86.5)32	Has
	(15.7)6	(13.5)5	Doesn’t have

**Fig 1:**
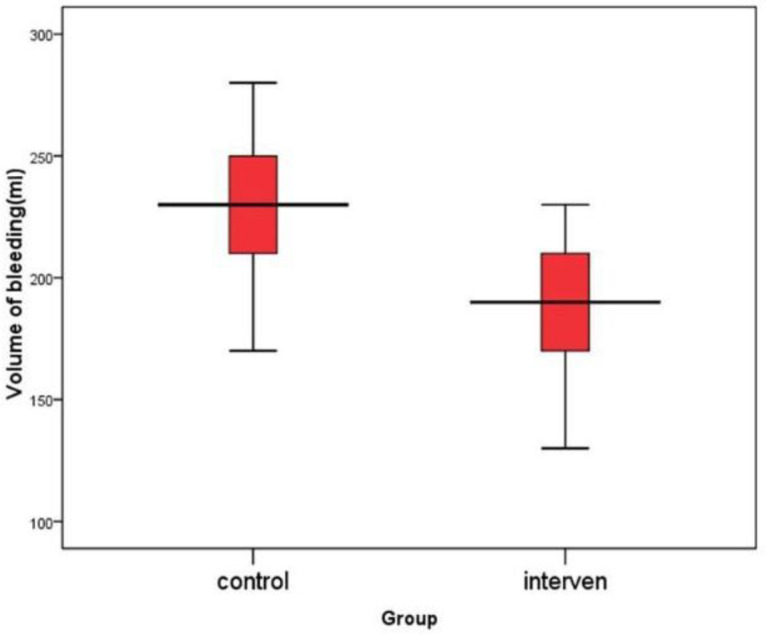
Volume of bleeding in the two groups of intervention and control

There was no statistically significant difference between patients with chronic rhinosinusitis undergoing endoscopic sinus surgery in the intervention and control group regarding hemoglobin, hematocrit, BT, CT, PT, INR, serum platelet, sodium, and potassium levels, and mean of arterial pressure. In patients with chronic rhinosinusitis undergoing endoscopic sinus surgery in the intervention group, the mean intraoperative bleeding volume was significantly lower than the control group (P <0.001), and the mean time of surgery was less than the control group (P <0.001) (Fig 1). 

**Fig 2:**
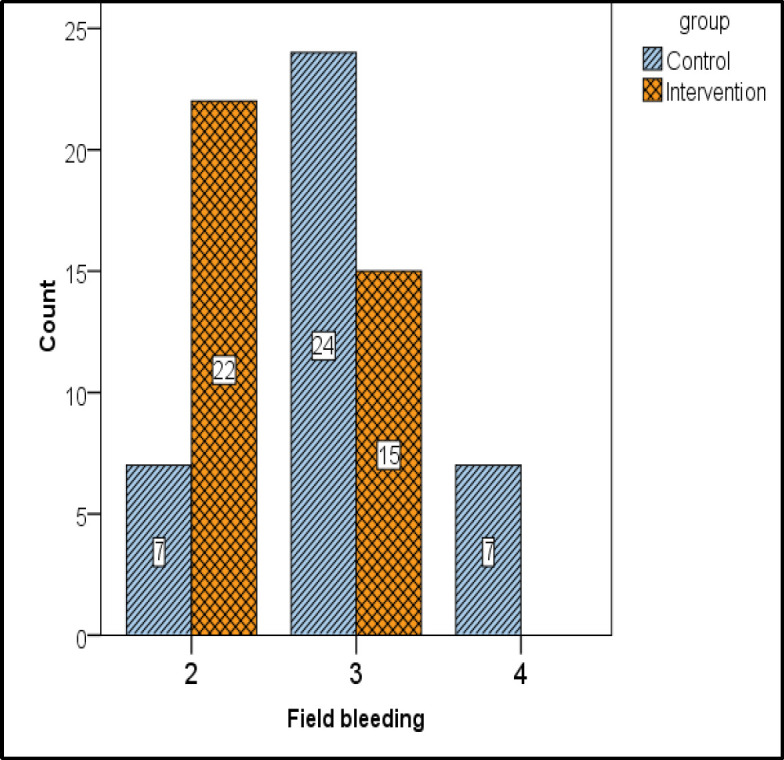
The mean score of surgical field quality in the two groups of intervention and control

 The mean score of surgical field quality was 2 in the intervention group and 3 in the control group. Based on the non-parametric statistical results of the Mann-Whitney test and Boezaart scale, there was a significant difference (P <0.001) between patients with chronic rhinosinusitis undergoing endoscopic sinus surgery in the intervention and control groups in terms of the mean score of surgical field quality, intraoperative bleeding volume (Fig 2).

Also, in patients with chronic rhinosinusitis undergoing endoscopic sinus surgery in the intervention group with polyps, the mean surgery time and the bleeding volume were significantly (P <0.001) less than in patients without polyps (Table 2).

**Table 2:** The mean of surgery time and the bleeding volume in the intervention group with and without polyps

**P-value**	***Intervention*** *** group (polyp)***		**Variable **
	**Doesn’t have (n=24)** **Mean±sd**	**Has (n=24)** **Mean±sd**	
>0.001	118.84±10.03	99.56±12.15	Time of surgery (minute)
>0.001	208.46±12.14	176.46±22.58	Bleeding rate (ml)

## Discussion

In this clinical trial, 75 patients with chronic rhinosinusitis were selected for endos- copic sinus surgery and randomly assigned to two groups, topical furosemide (intervention) and normal saline (control). It means that prescribing topical nasal furosemide spray at a dose of 20 micrograms twice a day one week before surgery significantly reduces the bleeding during surgery, improves the quality of the surgery field, and reduces the surgery time in patients with chronic rhinosinusitis. Furthermore, the effect is more sensible in patients with polyps than others. Also, no complication such as electrolyte disorders was observed by using this drug. Furosemide is a loop diuretic and inhibitor of lC2-KaN cotransporter. Furosemide reduces prostaglandins E2 and F2a by the mechanism of reducing the secretion of arachidonic acid and inhibits the release of cytokines. Inhaled furosemide does not have serious complications or diuretic effects (10). At the epithelial level of the respiratory system, furosemide reabsorbs sodium, followed by water, resulting in shrinkage of the polyp. Therefore, the effects of furosemide on reducing edema and inflammation and thus reducing the size of the polyp can reduce bleeding, especially in patients (11). In this study, to observe the effect of topical furosemide on the amount of bleeding and the quality of the surgical field, according to the time of furosemide effect, patients used topical furosemide spray twice a day one week before surgery. In a study conducted by Baradaranfar et al. in 2016 on the efficacy of topical furosemide and oral corticosteroids in controlling bleeding during endoscopic sinus surgery, there was no significant difference between the group using furosemide and corticosteroids in terms of bleeding mean, quality of surgeon's vision and surgery time (8). However, in our study, due to contraindication of using corticosteroids considering the inclusion criteria of the research, the effect of topical furosemide was compared with saline instead of oral corticosteroids, and a significant difference was seen between the group receiving furosemide and the group of saline in bleeding rate, mean score of surgeon's visual quality. Furthermore, the difference was more significant in patients with polyps, indicating the effect of topical furosemide on intraoperative bleeding and better quality of the surgical field; the result confirms the opinion of Baradaranfar’s research on the effectiveness of furosemide spray. 

In a clinical trial conducted by Hashemian et al. in 2016, the effect of topical furosemide was studied on the recurrence of nasal polyps after endoscopic sinus surgery, and the results showed a significant effect of prescribing topical furosemide on the reduction of polyp size after endoscopic sinus surgery, which showed the effect of the drug on inflammatory cells compared to placebo. Also, no significant difference was observed between the groups receiving furosemide spray and placebo in terms of the complications of nasal irritation, headache, and congestion (11). In our study, instead of the effect of topical furosemide on reducing the recurrence and severity of polyps, it was studied to improve the quality of the endoscopic surgical field and reduce intraoperative bleeding. In our study, no significant complications of furosemide spray were reported by patients, and no electrolyte disorders were observed in the tests indicating 

the safety and security of topical furosemide. Also, the efficacy of furosemide in patients with polyps was significantly higher than in patients without polyps, which seems to be due to the effect of furosemide on inflammatory cells.

In a study by Kroflic et al. in 2006, the effect of oral prednisolone and topical furosemide spray were compared one week before endoscopic sinus surgery. The study showed that furosemide spray did not affect inflammatory cells but reduced tissue edema (7). The present study evaluated bleeding during surgery and quality improvement of the surgical field in people with and without polyps, which were reduced compared to the control group. Moreover, the reduction was more significant in patients with polyps, which could indicate the effect of this drug on inflammatory cells and result in a reduction of tissue edema. The quality of the surgical field of patients receiving furosemide spray was better in our study, and their Boezaart score was lower than that of patients receiving spray furosemide in Kroflic et al. In the present study, instead of prescribing corticosteroids, the effect of furosemide spray was studied on intraoperative bleeding; considering its good efficacy, it can be used as a low-complication option to reduce bleeding and improve the quality of the endoscopic sinus surgical field.

## Conclusion

prescribing nasal furosemide spray at a dose of 20 micrograms twice a day one week before the start of endoscopic sinus surgery in patients with rhinosinusitis significantly reduces the amount of bleeding during endoscopic sinus surgery and the time of surgery. It improves the quality of the surgical field. 

Reduction of bleeding and time of surgery in patients with polyps is more sensible than in patients without polyps, and no complications such as electrolyte disorders were observed in using the drug. The low sample size was a limitation of the present study. Performing similar studies and comparing nasal furosemide spray with other drugs, short-term and long-term complications of using nasal furosemide spray can make this drug a choice in reducing 

the size of polyps and thus reduce intraoperative bleeding and preventing recurrence.
